# GANN: Genetic algorithm neural networks for the detection of conserved combinations of features in DNA

**DOI:** 10.1186/1471-2105-6-36

**Published:** 2005-02-22

**Authors:** Robert G Beiko, Robert L Charlebois

**Affiliations:** 1 Institute for Molecular Bioscience, The University of Queensland, Brisbane 4072, Australia; 2 Department of Biology, University of Ottawa, Ottawa, ON, K1N 6N5, Canada; 3 Genome Atlantic, Department of Biochemistry and Molecular Biology, Dalhousie University, Halifax, NS, B3H 1X5, Canada

## Abstract

**Background:**

The multitude of motif detection algorithms developed to date have largely focused on the detection of patterns in primary sequence. Since sequence-dependent DNA structure and flexibility may also play a role in protein-DNA interactions, the simultaneous exploration of sequence- and structure-based hypotheses about the composition of binding sites and the ordering of features in a regulatory region should be considered as well. The consideration of structural features requires the development of new detection tools that can deal with data types other than primary sequence.

**Results:**

GANN (available at ) is a machine learning tool for the detection of conserved features in DNA. The software suite contains programs to extract different regions of genomic DNA from flat files and convert these sequences to indices that reflect sequence and structural composition or the presence of specific protein binding sites. The machine learning component allows the classification of different types of sequences based on subsamples of these indices, and can identify the best combinations of indices and machine learning architecture for sequence discrimination. Another key feature of GANN is the replicated splitting of data into training and test sets, and the implementation of negative controls. In validation experiments, GANN successfully merged important sequence and structural features to yield good predictive models for synthetic and real regulatory regions.

**Conclusion:**

GANN is a flexible tool that can search through large sets of sequence and structural feature combinations to identify those that best characterize a set of sequences.

## Background

The minimal requirement for transcriptional activation is recruitment of an RNA polymerase complex to a promoter sequence of DNA upstream of an open reading frame (ORF). Most genes are also potentially under the control of DNA-binding regulatory proteins or *transcription factors *that can activate or silence transcription. In bacteria, activator and repressor proteins bind to operator sequences that are typically found near the promoter, and promoter specificity is typically conferred through the sigma subunit of RNA polymerase, which binds the promoter directly [1]. Eukaryotic transcription factors interact with DNA within the promoter, and are responsible for recruitment of the RNA polymerase complex [2]. Regulatory proteins also bind to conserved sites near the promoter region, as well as to enhancers that can be far (> 10 000 nucleotides) upstream or downstream of the promoter. In all domains of life, transcription factors that bind near the promoter are typically involved in either stabilizing or disrupting the initiation of transcription, while distal enhancer sequences are needed to destabilize the nucleosomes that usually prevent the initiation of transcription in eukaryotes [3].

The identity and spacing of these protein binding sites are key contributors to the responsiveness of a gene to changing cellular conditions, and sets of genes or operons that are expressed under similar conditions often have similar sets of regulatory elements in their 5' upstream regions. Recent programs such as ClusterBuster [4] and Promoter2.0 [5] recognize the need to detect combinations of binding sites in order to characterize promoters or whole regulatory regions. An additional challenge is the possibility of multiple cluster types within a class of genes that respond to the same regulatory stimulus. Artificial neural networks (ANNs) are suited to the task of discovering complex interactions within a set of features, and identifying multiple alternative solutions that yield the same type of response. This type of problem is not *linearly separable *[6], and would apply to classification problems such as the regulatory cascade of genes that is induced by lipopolysaccharide in mammals, where the entire set of genes is upregulated in response to a stimulus, but time or subresponse specificity is conferred by one of a set of regulatory modules [7,8].

Another issue in the detection and modelling of regulatory regions is the assumption of *additivity *in DNA-protein interactions when position-specific scoring matrices (PSSMs) are used to model binding sites. In fact, binding affinity has been shown in some cases to depend on interactions between sites [9-11], which suggests that more-sophisticated modelling schemes may be necessary to build accurate models of binding site affinity. PSSMs are a useful and versatile tool and may be adequate for binding site modelling in many cases (see for instance [12]), but the additional flexibility of ANNs may be useful in representing non-additive relationships among components of a binding site.

The genetic algorithm (GA) is a powerful tool for combinatorial problems of model optimization and feature selection when the 'model space' is complex and has many local optima. Genetic algorithms carry out a number of simultaneous searches in model space, with one or more recombination operators to periodically combine the results of two or more searches, permitting large scale 'jumps' out of locally optimal regions. GAs have been applied to several tasks in computational biology, including sequence alignment (SAGA: [13]) and phylogenetic inference (MetaPIGA: [14]). ModuleSearcher [15] is a recent application of genetic algorithms to the problem of *cis*-regulatory module detection, with the stochastic GA approach shown to yield similar outcomes to an exact search method in substantially less time. ClusterScan [16] is another recent approach that uses genetic algorithms to detect optimal combinations of binding sites from the TRANSFAC database [17].

Another important question in modelling regulatory regions involves the representation of binding sites. While position-specific scoring matrices (PSSMs) are a popular and effective way of representing conserved sites [18], other strategies such as consensus sequences, sequence composition and structural features [19] can be considered as well. Structural features should be of particular interest, since DNA deformability appears to play a role in at least some regulatory interactions such as the binding of *Escherichia coli *integration host factor (IHF) to its target sequence [20], the correct orientation of both halves of bacterial promoter sequences [21] and the dynamics of histone/DNA interactions [22]. DNA structural properties have been derived from crystal and nuclear magnetic resonance experiments and from theoretical simulations, and different oligonucleotides have different propensities toward unwinding, wrapping around other molecules, and deformation in response to ligand binding (reviewed in [23]). While there is still considerable controversy in the structural field about issues such as A-tract curvature [24], and there have been questions about the role of experimental conditions in determining results [25-27], carefully selected parameters can permit the testing of specific structural hypotheses pertaining to regulatory protein-DNA interactions.

We have developed GANN, a software suite that uses machine learning methods to identify combinations of the features listed above that best distinguish between a positive set (containing, for instance, a set of regulatory regions from co-expressed genes) and a negative set. GANN implements all of the binding site representations described above, allowing examination of models of different complexity as warranted by the type of binding site modelled and the amount of training data that is available.

## Implementation

**Figure 1:**
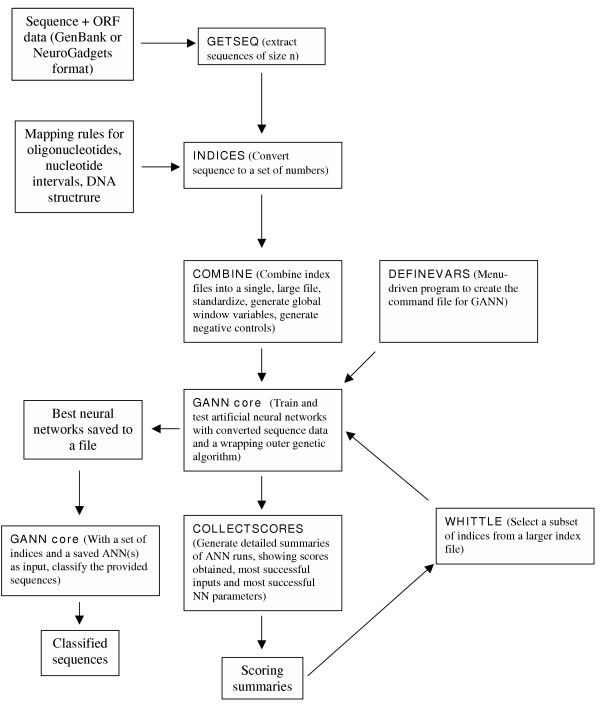
Programs and data flow for GANN.

GANN contains a set of programs for sequence extraction, retrieval and grouping of requested patterns, neural network analysis of these patterns and collection of results. Each program in the suite can accept either the output of the previous program, or an appropriate set of data generated from an external method. The components of GANN and the flow of data through the system are shown in Figure 1. 'Indices' and the core machine learning component are implemented in C++, while the other programs that read, interpret and combine text files are written in Perl.

### Sequence extraction

The first program (GetSeq) reads in either a GenBank file of annotated genome sequence, or raw sequence and a list of open reading frames (ORFs) of interest generated with the NeuroGadgets Inc. Bioinformatics Web Service [28]. GetSeq identifies and extracts upstream intergenic regions of a specified length and labels them as the positive set, and can also extract negative set sequences from intergenic regions that are not immediately upstream of ORFs, or directly from the protein-coding regions.

### Generation of sequence and structure indices

The Indices program takes as input a set of positive and negative set sequences (such as those generated by GetSeq), and can compute various properties of the sequences. The input sequences can be subdivided into overlapping windows of any size prior to the calculation of index values. The following indices can be calculated:

- Oligonucleotide frequencies are computed by counting the number of instances of a given *k*-mer within a window, then dividing by the length of that window. The program can determine the frequency of all *k*-mers of a specific length, or can assess any user-specified set of *k*-mers, which may include IUPAC notation to represent degenerate nucleotides.

- User-specified PSSMs can be counted for each sequence window. The user provides a set of scores for each type of nucleotide at each position within a PSSM, and a threshold score. The program will then count and record the number of sequence instances within each window that yield a PSSM score greater than the specified threshold.

- Structure and flexibility rules are implemented via a text file, by assigning floating-point values to each *k*-mer of a given length. The average score for a given sequence window is then computed by adding the scores for each overlapping *k*-mer within the sequence, and dividing by the total number of *k*-mers considered. Any numeric encoding of a complete set of *k*-mers can be specified: features sampled from publications such as [29-31] are available at the GANN website (see below).

After the extraction of indices, the Combine program merges the different index files into a single large file that is used as the input for the machine-learning software. Combine also allows the computation of Z-scores, thus representing each index value in terms of the number of standard deviations from the mean, and can identify peak values for a given index across a set of windows. Combine randomly subdivides the positive and negative index sets into training and test sets, and can also generate a negative control by randomly reassigning some positive and negative set members to the opposite category, yielding a disruption of patterns that were previously consistent within a single set. This type of control sets a 'baseline' for classification accuracy that can be compared to real experimental results.

### Pattern classification

The core of GANN is the neural network classification system. The indices generated from 'Indices' and 'Combine' are presented as input to an artificial neural network, which is trained with either backpropagation or a genetic algorithm to maximize the discrimination between the positive and negative sets. Since the number of indices associated with each sequence is potentially very large, the Outer Genetic Algorithm (OGA) presents random subsets from the pool of indices to a series of neural networks. The unit of selection for the OGA is a 'Chromosome' that contains a predetermined number of indices sampled from the larger pool, and a set of parameters that define the architecture and connectivity of the ANN. The constitution of a population of OGA Chromosomes is determined randomly in the first generation, with random sampling of indices from the pool and ANN parameters sampled randomly from within a set of ranges specified by the user. Each OGA Chromosome is used to construct an ANN, which is then trained to yield optimal predictive accuracy on the training set defined by the Combine program above. At the end of training, performance on the test set is evaluated, and the fitness of the OGA Chromosome is equal to its predictive accuracy on the test set samples. The predictive accuracy is defined as follows:


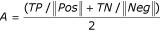


Where TP and TN are the number of correctly classified positive and negative test set examples, and ||*Pos*|| and ||*Neg*|| the size of the positive and negative test sets, respectively. This formula assigns equal weight to the positive and negative sets regardless of their size, so the ANN cannot achieve an artificially high score by predicting every case as a member of the larger (training or test) set.

The OGA Chromosomes with highest fitness are then permitted to 'recombine', yielding new subsets of indices that are trained in the same manner, while less successful indices are gradually lost from the population. The classification potential of indices can be evaluated by examining the scores of neural networks that include these indices in their input set, and through a 'population genetics' approach that traces the frequency of indices through several rounds of OGA recombination and selection.

Training of the neural networks is performed using either backpropagation of errors [32] or with an 'inner' genetic algorithm (IGA). An IGA Chromosome consists of a set of floating-point values, each representing a connection weight within the ANN that is being trained. The fitness of each IGA Chromosome within a population is equal to the predictive accuracy of the specified ANN as defined in the equation above, but on the *training *set. IGA Chromosomes with relatively high fitness are then subjected to stochastic recombination and mutation of parameters to yield a new population of Chromosomes that are used in the next round of training. While gradient-descent training methods for ANNs (such as backpropagation) can easily get trapped in local optima of the solution space, the recombination option of genetic algorithms permits a search to 'jump' through the solution space and escape local optima. The optimisation of network architecture and connection weights is similar to the 'structure evolution' method of [33], which has been applied to problems of biological pattern detection [34,35], but our method differs in the partitioning of the connection weight and architecture components of the optimisation.

There are many variables within GANN whose values can be specified by the user. While most default settings will be adequate in most situations, parameters such as the number of feature combinations generated by the OGA and the number of features in each combination should be chosen carefully. The 'DefineVars' program provides a set of menus that allow the user to set these parameters and write them to a configuration file that is input to GANN.

In addition to reporting the scores of trained artificial neural networks, GANN will save information about the topology, connection weights and constituent indices of each neural network instance that achieves a generalization score above a specified minimum threshold. If GANN is invoked with any of these saved neural networks as input in addition to a table of indices, then it will use the input neural network to classify the new table. This process can yield functional predictions for sequences whose true classification is unknown.

## Results and discussion

Two sets of detection experiments, both based on DNA sequences extracted from the *Escherichia coli *K12 genome, are presented to illustrate the performance of GANN. Both of these experiments included a set of 250 nucleotide sequences, each 100 nucleotides (nt) in length, which were extracted from between convergently transcribed genes in the *E. coli *genome using the GetSeq program. These sequences were chosen because they are intergenic like upstream regulatory regions and not subject to the evolutionary constraints of protein-coding sequences, but are not expected to contain functional transcriptional regulatory features since they are exclusively 'downstream' of one gene in each direction. In the first experiment, we created an artificial positive set by inserting conserved sequences into a subset of the 250 sequences, with the remainder constituting the negative set. The entire set of 250 sequences was used as the negative set in the second experiment, while the positive set consisted of 212 upstream regulatory regions containing experimentally validated binding sites for the σ^70 ^protein of *E. coli*.

Several run parameters were consistent across both experiments. Each OGA Chromosome contained a total of 8 indices, to allow the simultaneous representation of several sequence and structure properties. The population size (= number of OGA Chromosomes) was determined by multiplying the total number of indices by 10, then dividing by the number of indices (8) per OGA Chromosome. This formula ensured that indices would be represented 10 times each on average in the initial randomly generated population, and 99.9% of all indices should occur at least 3 times in the population according to the Poisson distribution. Thirty rounds of OGA Chromosome evaluation and selection were performed in each run. While GANN can evolve ANN architecture and learning parameters as well as combinations of indices, we chose reasonable ANN parameters (available at the GANN website) and fixed them for the entire run.

The performance of different sets of indices was expressed in terms of the predictive accuracy (= score on the test set of sequences) described above. Differences in predictive accuracy are expected across replicates, because random partitioning of sequences into training and test sets is likely to yield variation in the frequency of some features that do not define the whole set. However, average predictive accuracy can be estimated by taking the mean across replicates. Indices that are retained in every replicate of an experimental run are more likely to reflect true characteristics of the sequences under consideration, though the redundancy of many indices (different window sizes, different percentiles for PSSM scores, and correlated frequencies of some *k*-mers) may yield multiple alternative solutions that are equally good. A final indicator of index performance is the composition of OGA Chromosomes that yield high predictive accuracy: if a set of features is important for characterization of a set of sequences, then each of those features should be represented by at least one index in the best OGA Chromosomes.

### Experiment 1 – Synthetic positive set

In the first experiment, the positive set was constructed by adding conserved features to 76 of the 250 sequences (~30%) described above. Each member of the positive set was modified by adding a nucleotide decamer with high conformational mobility (CM). Five thousand unique decamers were generated randomly, and each of these was assigned a CM score based on the dinucleotide table in [30]. Decamers from this set that scored in the top 5% of all CM values were selected at random to be added to members of the positive set. One of two types of conserved binding site was also added to each member of the positive set. The set of experimentally validated binding sites for cAMP receptor protein (CRP) and leucine-responsive regulatory protein (Lrp) were extracted from RegulonDB [36], and each positive set sequence gained a binding site randomly selected from one list or the other.

**Figure 2:**
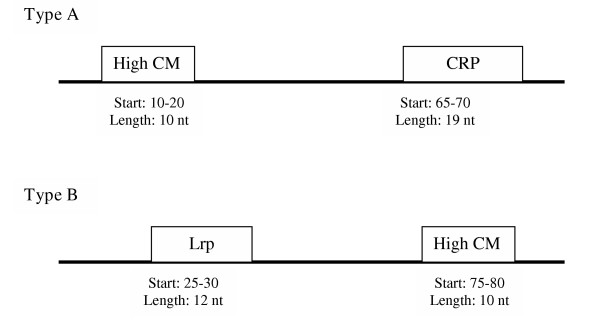
Construction of the synthetic positive set of sequences in Experiment 1. The range of starting positions and length of each added feature are indicated (CM = conformational mobility, CRP = cAMP receptor protein, Lrp = leucine-responsive regulatory protein).

The construction of synthetic conserved regions is summarized in Figure 2. 'Type A' positive set sequences consist of a high CM decamer beginning anywhere between positions 10 and 20, and a randomly chosen CRP binding site of length 19 that starts between positions 65 and 70. The order of patterns is inverted in the 'Type B' sequences, with the 12 nt Lrp binding site beginning between positions 25 and 30, and the high CM decamer starting anywhere between positions 75 and 80. A total of 34 Type A and 42 Type B sequences were generated.

Once the positive and negative set sequences were obtained and assembled, the Indices program was used to extract several different types of information from them, with varying window sizes depending on the features being examined. The overlap between adjacent windows was chosen to be 50% of the window length, so if a window of size 10 covered sites 1–10 in a sequence, the next window would cover sites 6–15.

- PSSMs for Lrp and CRP binding sites were constructed from the set of binding sites in RegulonDB. Frequency matrices for each site were constructed by dividing the number of occurrences of each residue at each site by the total number of sites (72 Lrp, 128 CRP). These frequencies were then divided by the 'background' frequency of each corresponding nucleotide in the set of intergenic sequences. Since the background frequency of each nucleotide was within the range 0.250 ± 0.005, background frequencies of 0.25 were assigned to each nucleotide. The PSSM was then obtained by taking the natural logarithm of each value in the corrected frequency matrix. Threshold values for PSSM predictions were determined by scoring 50 000 random sequences of the appropriate length against the PSSM, and identifying the scores that corresponded to the 99^th^, 95^th^, 90^th^, and 80^th ^percentiles. The number of sequence matches above each PSSM threshold was computed for windows of size 20 and 40.

- The conformational mobility of sequence windows of size 10 and 20 was computed according to the dinucleotide values in [30].

- Counts of all *k*-mers of size 1 (mononucleotides) and size 2 (dinucleotides) were computed for windows of size 10 and 20.

The computations described above yielded a total of 450 indices: 360 describing *k*-mer counts, 64 describing the counts of PSSM 'hits' at different thresholds, and 36 describing the conformational mobility of different windows of sequence. These indices were then combined in four different ways to yield separate tests of different subsets. The *k*-mer frequencies were included in every set as a 'background' measure of predictive power with no explicit hypothesis. Set 1.1 included only the *k*-mer frequencies, while set 1.2 added the indices of conformational mobility, set 1.3 included the PSSM scores, and set 1.4 included all three types of index. We initially performed runs where index values were not standardized, but found that indices with values that were not close to zero, particularly the CM indices which ranged between 40 and 70, did not perform well and were consistently eliminated from the population of OGA Chromosomes. In response to this, we standardized all indices for the experiments described below and in Experiment 2.

Set 1.4, with the full set of 450 indices, was used to define the number of OGA Chromosomes. The formula at the beginning of this section yielded a recommendation of 562.5 OGA Chromosomes per generation, which was rounded up to 600 and applied to all four sets. 'Combine' was used to randomly subdivide the positive and negative set sequences into training and test sets with a ratio of 2:1. This random reassignment was repeated five times, and five corresponding negative control sets were generated as described in *Generation of Sequence and Structure Indices *above.

**Figure 3:**
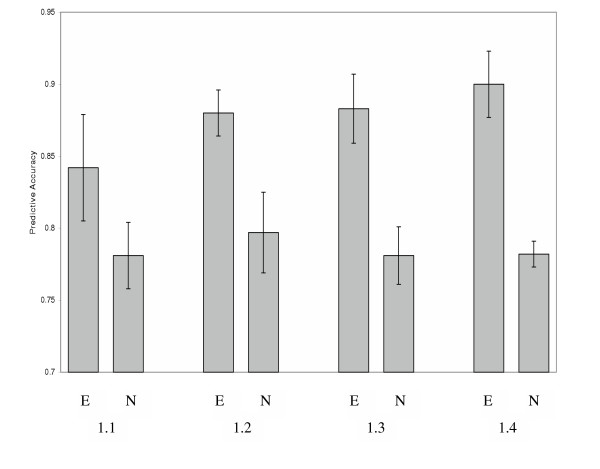
Maximum predictive accuracy of OGA Chromosomes on the generalization set in Experiment 1. Mean ± standard deviations are shown for the five replicates of the experimental (E) and negative control (N) runs for the four data sets (1.1 to 1.4) used.

The mean of the best generalization scores achieved in each replicate over 30 rounds of OGA evaluation and selection is shown in Figure 3. The 4 groups of negative control runs corresponding to the four data sets all yielded a mean best score between 0.78 and 0.79, and the range of scores in each case did not overlap with the range of the corresponding five experimental replicates. However, the average generalization score of experimental set 1.1 was only 5–6 % higher than the corresponding negative control runs. Set 1.2, which included CM as well as *k*-mer counts, yielded a mean generalization score of 0.880, a substantial improvement over set 1.1 with no overlap in the range of maximum scores between the two sets. Set 1.3, which considered *k*-mer counts and PSSM scores at several thresholds, yielded a mean best generalization score of 0.883, which was substantially better than set 1.1 and indistinguishable from set 1.2. Finally, set 1.4 yielded a small improvement over sets 1.2 and 1.3 in generalization score, with a mean of 0.900. These results suggest that the inclusion of PSSMs and flexibility indices yielded a substantial increase in predictive accuracy over the background of *k*-mer counts, with the combination of the two possibly producing a further slight increase.

**Figure 4:**
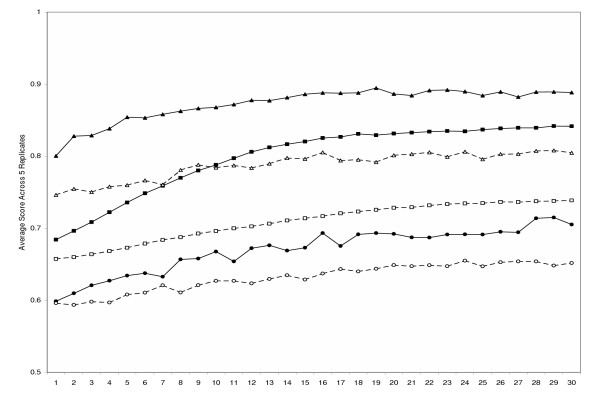
Change in the mean (squares), maximum (triangles) and minimum (circles) generalization scores for the 600 OGA Chromosomes in each of 30 training rounds for set 1.4. Plotted values represent the mean of each value across all 5 replicates of either the experimental (filled shapes, solid line) or negative control runs (empty shapes, dashed line).

Figure 4 shows the change in the mean, maximum and minimum generalization scores for the 600 OGA Chromosomes in each of 30 training rounds for set 1.4. The mean over all five replicated runs is shown for both the experimental and negative control runs. There is an upward trend with all six values, which shows that improvements in the mean performance are due to both the creation of new, advantageous combinations of indices by the OGA as evidenced by the increase in the maximum score, and through the elimination of bad indices, shown with the increase in the minimum score. The difference in mean generalization score between the experimental and negative control runs is very low (< 0.025) in the first OGA generation, but increases rapidly to 0.11 – 0.12 within the first ten generations of optimisation. This trend is consistent with the idea that many indices in the experimental runs are not good at distinguishing between the positive and negative sequence sets, and their replacement with more copies of good indices yields better predictive accuracy. However, poor indices are expected to persist to some degree through the population, since they can 'hitchhike' with good indices through many rounds of OGA training and may even increase in frequency if they are associated with an otherwise good combination of indices. The low (< 0.7) predictive accuracy of some experimental OGA Chromosomes in the last round of training may be due to recombination events that merge sets of hitchhiking indices.

If sets of indices that yield the best predictive accuracy are preferentially selected for recombination by the OGA, then good indices should increase in frequency with successive rounds of testing and recombination. Only five indices were present in the final population of all five replicated experimental runs of set 1.4: one measuring the count of sites scoring in the 99^th ^percentile of the CRP matrix between positions 60 and 99, another measuring the same quantity for Lrp sites between positions 20 and 59, an index describing the conformational mobility between sites 80 and 90, and two indices representing the count of GC dinucleotides and C mononucleotides between positions 29 and 39. The first three indices are easy to understand, as they correspond directly to features (binding sites and a high CM region) that were deliberately inserted into the positive sequence set. However, the third index is less clear until the Lrp sites from RegulonDB are examined in detail: these sites are G+C poor in general, and of the 72 × 11 = 792 dinucleotides contained in the full set of Lrp binding sites, only 20 of these are GC steps. If all 16 possible dinucleotides were present with equal frequencies, then the pair would be present 49 or 50 times, so GC is strongly underrepresented in this data set. Thus, it appears that the GC content in this region is included in many OGA Chromosomes because it is another indicator of the presence or absence of the Lrp binding site in this region.

### Experiment 2 – σ^70 ^positive set

The second experiment tested the ability of GANN to distinguish between the 250 unmodified intergenic sequences described earlier, and a set of sequences containing binding sites that are recognized by the 'housekeeping' σ^70 ^subunit of RNA polymerase in *E. coli*. The 212 promoter-containing sequences in the positive set were extracted from a larger set defined in [37]. Where multiple promoter sequences were identified in a single upstream region, one of these sequences was chosen at random for inclusion in the positive set. All sequences in this experiment were 100 nt in length, and the positive set sequences were aligned at the transcription start sites of the relevant promoters. The -35 box (consensus 'TTGACA') was typically contained between positions 40 and 49 in the sequence, while the Pribnow box (consensus 'TATAAT') was located approximately between positions 65 and 70 in the positive sequences.

Indices were constructed in a similar manner as in Experiment 1. Separate PSSMs for the -10 and -35 boxes recognized by σ^70 ^were constructed from 250 promoter sequences in the data set, again with background frequencies of 25% for each nucleotide. As with CRP and Lrp above, the 99^th^, 95^th^, 90^th ^and 80^th ^percentile scores were generated for the -10 and -35 boxes from a set of random sequences, though only 1000 random sequences were generated for each case. Since the two halves of the σ^70 ^consensus sequence are only six bases in length, indices based on PSSM matches for a window size of 10 nt as well as 20 nt and 40 nt were calculated. Indices of conformational mobility and *k*-mer frequency were calculated as in Experiment 1 above. A total of 618 standardized indices were generated in this experiment: 360 describing *k*-mer counts, 222 describing the counts of matches to the -10 and -35 PSSMs at different thresholds, and 36 describing the conformational mobility of different windows of sequence. Five sets of experiments were performed, with five experimental and five negative control runs in each. All sets (2.1 to 2.5) included the *k*-mer count indices, and these were the only indices considered in set 2.1. Sets 2.2 and 2.3 also included the -35 and -10 PSSMs respectively, while set 2.4 included both. All 618 indices were considered by set 2.5.

Runs were performed as in Experiment 1 above, with the exception of the OGA Chromosome population size. With 618 indices in total, the formula described at the beginning of this section yielded a recommendation of 772.5 OGA Chromosomes per generation, which was rounded up to 800 and applied to all five sets.

**Figure 5:**
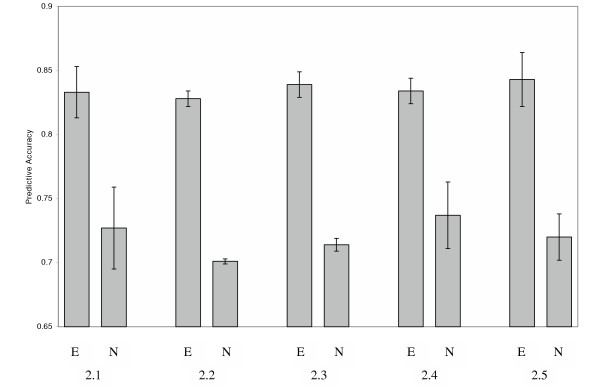
Maximum predictive accuracy of OGA Chromosomes on the generalization set in Experiment 2. Mean ± standard deviations are shown for the five replicates of the experimental (E) and negative control (N) runs for the five data sets (2.1 to 2.5) used.

The mean of the best generalization scores achieved in each replicate over 30 rounds of OGA evaluation and selection is shown in Figure 5. Remarkably, there was no substantial difference between any of the experimental sets, which yielded mean predictive accuracies between 0.828 for set 2.2 and 0.843 for set 2.5. This range was smaller than that of the predictive accuracies of the five negative control treatments, which ranged from 0.701 for set 2.2 to 0.737 for set 2.4. No clear trend exists for either the experimental or negative control sets, suggesting that the CM and PSSM indices did not yield any improvement in predictive accuracy over the *k*-mer counts alone, and that the inclusion of the σ^70 ^PSSMs did not yield a more-precise model of the promoter sequence.

While the predictive accuracy did not change across multiple sets of experiments, the type of indices that were selected by the OGA varied from set to set. The most successful index overall described the frequency of the dinucleotide 'TA' between positions 65 and 74, which corresponded to the position of the Pribnow box in the positive set sequences. This index was the only one present in all five replicates of sets 2.1, 2.3 and 2.4 after 30 rounds of OGA training, and was one of only three such indices for set 2.2. The other two indices that were retained by all five replicates of set 2.2 described the frequency of TA between positions 60 and 69, and the frequency of CA between positions 55 and 64. No single index was retained in all five replicate runs of set 2.5. In all sets that included PSSM representations, the appropriate PSSM component(s) were always retained in four out of five replicated runs. While PSSMs with a very high score threshold (99^th ^percentile) were favoured in Experiment 1, the PSSMs most commonly retained in the σ^70 ^experiments favoured lower percentile thresholds, with a roughly even distribution of indices representing the 95^th^, 90^th ^and 80^th ^percentiles. This effect may be due to greater degeneracy of σ^70 ^sites relative to the CRP and Lrp sites modelled earlier. Finally, four out of five replicate experimental runs of set 2.5 retained a CM index that covered positions 30–50 in the sequence. While no single index was retained in all five replicate runs of set 2.5, the OGA Chromosomes with the highest generalization scores all contained at least one instance of a PSSM for each of the two halves of the σ^70 ^consensus and a flexibility feature. Thus, more-selective indices such as those based on PSSMs were included in the majority of OGA Chromosomes when they were present in the experimental set, even if the gain in predictive accuracy was marginal.

## Conclusion

We have explored several features of GANN, most notably the ability to build classification rules for positive set members that form natural subsets, and the capacity to search through large sets of DNA sequence and structural indices to find combinations that yield optimal predictive accuracy. Our generalization accuracy of ~84% on the σ^70 ^promoter set is similar to the sensitivity of 86% and specificity of 85% reported by [38], though these results may not be directly comparable due to differences in the size of the data set and the definition of 'negative' examples. While our use of PSSMs in these experiments implies acceptance of the statistical mechanical theory of binding sites [39], GANN could also be used to build models that take into account interactions between individual residues within a binding site. The model thus constructed could then be compared against a traditional PSSM to see if better predictive accuracy is obtained on a test set of sequences. In focusing on the generation and testing of combinations of indices, we have not examined the performance of GANN when ANN architectural parameters are optimised alongside index combinations. One approach that avoids dealing with too many interactions at once can be to first use GANN to screen a large set of indices and generate a smaller list of indices with predictive power, and to perform a subsequent run where this smaller set of indices are examined in combination with variable ANN parameters.

In Experiment 1, we found that indices with mean values that are not close to zero should be standardized. A disadvantage of this approach is that standardization of a column of values is entirely sample dependent, since different finite samples from the same population of values will typically have different means and standard deviations, which may limit the accuracy of an ANN trained on one sample that is used to classify other subsamples from the same population. If standardization is to be applied, then there should be sufficient values in each index to yield a stable estimate of the population mean, as indicated with a low standard error. This is of particular concern since biological sequence samples often do not represent a random sample of all possible sequences, leading to biased estimates of sample mean and standard deviation.

The two primary goals of GANN are to allow multiple alternative representations of DNA features, and to permit the discovery of important combinations of these features through the hybrid genetic algorithm / neural network approach. A long-term goal is to use GANN to identify important combinations of motifs predicted from programs such as PatSer [40], which would permit the application of GANN to the task of identifying complex regulatory features in multicellular eukaryotes. The software packages are released under the GNU GPL and have been successfully tested and run on Win32 systems and on several flavours of UNIX. Complete documentation for GANN and the key files used in the experiments described in this manuscript are available at the project Web site.

## Availability and requirements

- Project name: GANN

- Project home page: 

- Operating systems: Win32, UNIX

- Programming language: C++, Perl

- Other requirements: none

- License: GNU GPL

- Any restrictions to use by non-academics: none

## Abbreviations

CM – Conformational mobility

GANN – Genetic Algorithm Neural Networks

OGA – Outer Genetic Algorithm

PSSM – Position-specific scoring matrix

## Authors' contributions

RGB was responsible for software design and implementation, and manuscript writing. RLC contributed to the design, and to the implementation of the genetic algorithm classes in C++.
